# Cannabidiol use and perceptions in France: a national survey

**DOI:** 10.1186/s12889-022-14057-0

**Published:** 2022-08-29

**Authors:** Clémence Casanova, Clémence Ramier, Davide Fortin, Patrizia Carrieri, Julien Mancini, Tangui Barré

**Affiliations:** 1 Aix Marseille Univ, Inserm, IRD, SESSTIM, Sciences Economiques & Sociales de la Santé & Traitement de L’Information Médicale, ISSPAM, Marseille, France; 2 Sorbonne Economics Centre, University, Paris 1 Sorbonne, Paris, France; 3 Public Health Department, APHM, BIOSTIC, Marseille, France

**Keywords:** Cannabidiol, Cannabis, France, Risk perception

## Abstract

**Background:**

Cannabidiol (CBD), a safe, non-intoxicating cannabis component, is growing in popularity in Europe and worldwide. However, CBD EU regulation is blurry, and consequent labelling and product quality issues may have implications for public health. There is therefore a need to assess the prevalence and perceived harmfulness of CBD use in EU countries, as well as to characterise CBD users. We aimed to do so in the French population.

**Methods:**

In December 2021, an online survey was conducted in a sample respecting the French adult population structure for key demographic variables. Sociodemographic, behavioural and CBD perception data were collected. Three separate regressions were performed to identify correlates of i) having heard of CBD, ii) using CBD, iii) perceived harmfulness of CBD. A hierarchical classification was also performed to identify profiles of CBD users.

**Results:**

The study sample comprised 1969 adults, of whom 69.2% had heard of CBD and 10.1% used it. Less than half (46.8%) of the former considered it harmful. Having heard of CBD was associated with younger age, being born in France, tobacco use, and cannabis use. CBD use was associated with younger age, tobacco use, cannabis use, poor self-reported general health status, and positive perception of alternative medicines. Cluster analysis revealed four different CBD user profiles based on socio-demographics and behavioural characteristics.

**Conclusion:**

Ten percent of the adults in this French study used CBD, and several user profiles emerged. Our results indirectly advocate clearer European CBD regulations to ensure safe and high-quality products.

**Supplementary Information:**

The online version contains supplementary material available at 10.1186/s12889-022-14057-0.

## Introduction

Cannabis contains a variety of cannabinoids, with Δ9-tetrahydrocannabinol (THC) and cannabidiol (CBD) being the most studied. The former is responsible for the ‘high’ provoked by cannabis use and is associated with a risk of dependence. In contrast, CBD does not have a high affinity for brain cannabinoid receptor 1 and is ‘non-intoxicating’ [1]. Although CBD may have anxiolytic properties [2] a World Health Organization review concluded that CBD “exhibits no effects indicative of any abuse or dependence potential”, has a good safety profile, and that “there is no evidence of recreational use of CBD or any public health-related problems associated with the use of pure CBD” [3]. A meta-analysis recently confirmed CBD’s safety profile [4].

Consequently, CBD-based products are proliferating worldwide. However, legislation on the use of cannabis-based products differs from country to country, even in Europe [5]. In France, CBD as a prescription drug (Epidyolex) is only available for ‘compassionate use’. CBD as a food supplement, such as THC-deprived dried cannabis flowers, or used as an ingredient is legal if THC levels remain below 0.3%. Most CBD products can be purchased in specialized shops, pharmacies, and supermarkets (dried cannabis flowers cannot currently be purchased in pharmacies and supermarkets). However, this legal framework is probably confusing for most French citizens (as shown in a UK-based sample [6]). Indeed, cannabis use, including for therapeutic purposes, is criminalized in France. Previous governments have restricted access to CBD products based on a 1990 Ministerial Order prohibiting cannabis use. In December 2021, the government banned the sale of CBD-rich THC-deprived cannabis flowers to consumers, but this ban was provisionally suspended in January 2022 by the Council of State, deeming it disproportionate to the product’s harmfulness [7].

CBD has a wide potential therapeutic spectrum [2]. There is evidence supporting its usefulness to treat epilepsy, as illustrated by the approval of Epidyolex. However, for other conditions including anxiety, pain/inflammation, schizophrenia, substance use disorders, and post-traumatic stress disorder, evidence from human studies is mixed. For most of these conditions, there is a lack of well-powered randomized, placebo-controlled studies to draw definitive conclusions [8]. In terms of real-world data, studies have highlighted potential benefits of CBD for overall quality of health and/or wellbeing, pain, depression, anxiety, and symptom improvement, especially in patients experiencing moderate to severe symptoms [9, 10].

CBD products are often marketed as complementary health products rather than medicines. There are concerns over the labelling of these products and their quality (i.e., THC levels, presence of contaminants) in terms of consumer safety and public health [11, 12, 13, 14]. 

Well-being has been highlighted as a major reason to use CBD in France and the UK [17, 18]. However, some consumers also use it to relieve disease symptoms [17]. Cannabis is generally perceived as less harmful than other psychoactive substances [19], and there is a decreasing trend in its perceived harmfulness [20, 21, 22]. As a cannabis-derived compound, CBD is likely to benefit from this change in perception. Moreover, CBD is increasingly seen as a ‘natural product’ [6]. In Canada and the US, a study has found that CBD users are more likely to perceive CBD as good for health than non-users [23]. Although CBD is commonly perceived as safe in Europe [6], few studies on the subject have been published to date. Moreover, to the best of our knowledge, data on the prevalence of CBD use at the national level in European countries are only available in Germany, where 4.3% of individuals have ever used CBD [24]. Studies conducted in non-representative samples of populations have reported a prevalence of CBD use ranging from 10.9% [6] to 14% [25] in Europe, 26.1% in the US and 16.2% in Canada [23].

Finally, as the harms caused by CBD may vary according to one’s health status and pattern of use, it is important to characterize the profile of CBD users. In Germany, lifetime CBD use was greater among educated, urban, tobacco (ex-)users [24] and similar results have been found in other settings [23]. These studies must be reproduced in other contexts and where CBD is legal, within sub-groups of CBD users. For instance, cannabis-CBD co-users may have different demographic characteristics compared to CBD only users [26]. It is therefore key to assess the extent of CBD use in EU countries, evaluate its perceived harmfulness, and characterize the profile of CBD users.

We aimed to assess the prevalence of CBD use in France, characterize the profile of users, and highlight factors associated with its perceived harmfulness from a large web-based national survey of French adults.

## Methods

### Design

The survey was conducted from 2 to 17 December 2021 as part of the SLAVACO research project [27]. SLAVACO is a multi-aim cross-sectional study. The aims were to collect data on psychoactive substance and CBD use, attitudes about the management of the COVID-19 pandemic, vaccination, and the place of scientific knowledge in related policy-making. Data were collected using self-administered online questionnaires.

### Study sample

The study population was comprised of a sample of the French population aged 18 years old and above. Participants were randomly selected from an existing online research panel, which included over 750, 000 nationally representative households (Bilendi SA®). The representativeness of the survey sample in terms of gender, age, type of professional occupation, and population density in the region of residence, was ensured using quota sampling, and respecting the adult French population structure (as per official census data). Participants were first contacted by e-mail, and enrolment continued until the necessary proportions were reached in the majority of quotas. To counterbalance any possible over- or under-representation of specific population categories, weighting factors derived from the National Institute of Statistics and Economic Studies (INSEE) data were used.

### Data collection

After obtaining informed consent, the web-based survey collected sociodemographic data including gender, age, city size (4 options), region (12 options), socio-professional category (8 options), highest educational diploma obtained (14 options), net household monthly income (7 range options), number of dependent children, difficulty paying bills (very easy, easy, difficult, very difficult), and country of birth.

The questionnaire also collected behavioural data, including frequency of tobacco, alcohol, cannabis use (do not want to answer, never, less than once a week, about once a week, several times a week, every day or almost every day) and preferred means to obtain information in general (7 options).

Three questions focused on perceptions about health issues; the first two were taken from the Minimum European Health Module and regarded self-perceived general health status and the presence of a chronic condition [28]. The third question examined respondents’ level of agreement with the statement: ‘alternative medicines provide better solutions to health problems than conventional medicines’ (do not want to answer, no opinion, fully agree, somewhat agree, neither agree nor disagree, somewhat disagree, fully disagree).

### Inclusion and exclusion criteria

Inclusion criteria were being an adult (≥ 18 years old) and residing in metropolitan France. Exclusion criteria were having missing data and answering ‘do not want to answer’ to the questions regarding ‘having heard of CBD’ and ‘CBD use’.

### Outcomes

Outcomes were based on the three following questions: i) “Have you ever heard of CBD?” (do not want to answer, had never heard of CBD, only knew the term ‘CBD’, knew a little about CBD, had good knowledge of CBD, have very good knowledge of CBD), ii) “Do you consume CBD-based products (oil, capsule, vaping etc.)?” (do not want to answer, never, less than once a week, around once a week, several times a week, every day or almost every day), and iii) “Do you think that CBD is harmful for health?” (do not want to answer, no opinion, not at all, slightly harmful, quite harmful, very harmful). The latter two questions were asked only to participants who did not answer ‘had never heard of CBD’. Participants who responded ‘had never heard of CBD’ were classified as non-users.

The first outcome was ‘having heard of CBD’ (vs. ‘never having heard of CBD’). The second outcome was ‘using CBD’ (vs. ‘never using CBD’). The third outcome was ‘perceiving CBD as harmful’ (i.e., from slightly harmful to very harmful) or ‘perceiving CBD as harmless’ (i.e., not at all harmful) (vs. ‘no opinion’).

### Explanatory variables

Current tobacco and cannabis use were dichotomized into yes vs. no. Alcohol consumption was categorized into never, occasional (less than once a week or around once a week) and regular (several times a week or every day or almost every day). Self-reported general health status was categorized as good, moderately good or poor. Answers to the statement that alternative medicines provide better solutions to health problems than conventional medicine were categorized into agree (‘fully agree’, ‘somewhat agree’), disagree (‘somewhat disagree’, ‘fully disagree’) and no opinion (‘neither agree nor disagree’). Participants who answered “do not want to answer” to this question were considered to have missing data.

The following variables had modalities merged according to frequencies in the population: socio-professional categories, educational level, number of dependent children and difficulties in paying bills.

### Statistical analyses

Study sample characteristics were compared according to the second outcome (non-user vs. user). Characteristics of participants excluded because of missing data were compared with those of included participants. Chi-squared tests and Student’s t-tests were used in these comparisons for categorical and continuous variables, respectively. The prevalence of CBD use was compared between response modalities for each descriptive variable, and Bonferroni corrections were applied to adjust for the higher risk of a type 1 error.

Three separate regressions were performed to identify correlates with the outcomes: two binary logistic (first and second outcomes) and one multinomial (third outcome) regression. An ascending hierarchical classification (AHC) was also performed to identify CBD user profiles.

Associations were assessed using odds ratios (OR) for the two logistic regressions and relative risk ratios (RRR) for the multinomial regression. Only variables with a liberal *p*-value < 0.20 in the univariable analyses were considered eligible for the multivariable models. The final multivariable models were built using a backward stepwise selection procedure. The likelihood ratio test (*p* < 0.05) was used to define the variables to keep in the final model.

For national estimates and for the analyses on the two first outcomes, data were weighted to correct for over- or under-representation of specific population categories with respect to gender, age, type of professional occupation and population density in the region of residence. Weighting factors were obtained using the Stata command *calibrate* with the logistic method, based on INSEE data.

A sensitivity analysis was performed for the perception outcome, by reclassifying respondents who answered ‘slightly harmful’ for the relevant question to the ‘harmless’ group.

The AHC was performed on the CBD user sub-sample, based on the set of variables identified in the second multivariable logistic regression model (CBD user vs. non-user), and the frequency of CBD use. For ease of interpretation, some variables were dichotomized before running the AHC. Gender was entered into the set of variables irrespective of its *p*-value in the regression model. The AHC involved two steps. First, a multiple component analysis was run. Data were then clustered using Ward’s method for cluster analysis. The choice of the number of groups to retain was based on the Duda-Hart rule [29]. Descriptive statistics of the groups were then provided, and Pearson’s correlations between variables used for the classification were assessed.

All analyses were performed with Stata software version 17.0 for Windows (StataCorp LP, College Station, TX).

## Results

### Study population characteristics

**Fig. 1:**
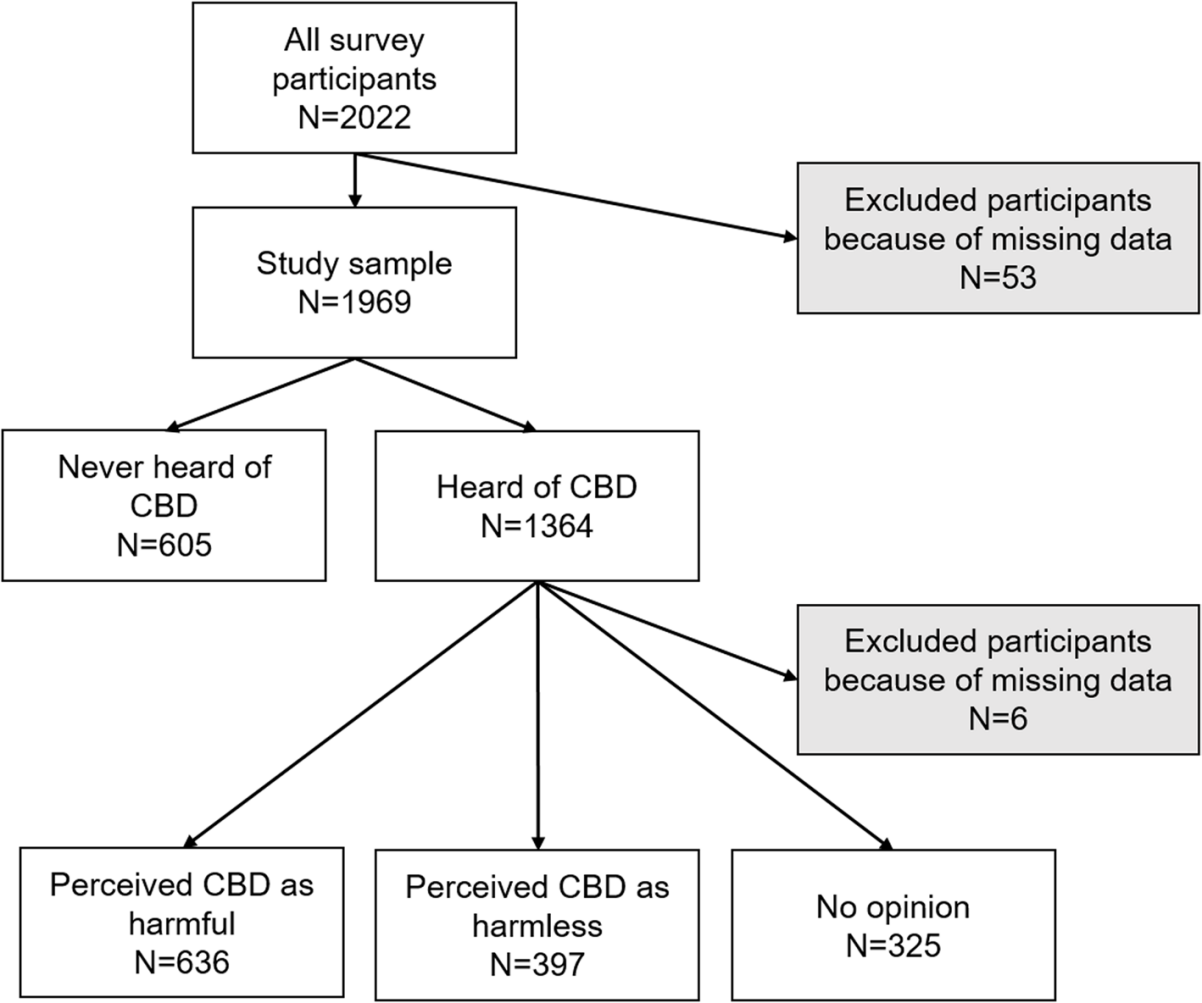
Flow chart of the study sample

The study population comprised 1969 participants (Fig. 1) whose characteristics are presented in Table 1. Differences between included and excluded participants (2.6%) were not substantial, and the impact of the weighting factors was marginal (Supplementary Table 1). Women comprised 52.4% of the study population, and mean age was 51.7 years. Over two thirds (69.2%, 95% confidence interval (CI) [67.1 – 71.3]) of the study population had heard of CBD, and 10.1% (95% CI [8.8 – 11.5]) used it (5.1% less than once a week, 1.7% around once a week, 1.7% several times a week, 1.6% every day or almost every day). Of those who had heard of it, 46.8% (95% CI [44.2 – 49.5]) considered it harmful (23.9% no opinion, 29.2% not at all harmful, 28.1% slightly harmful, 12.6% quite harmful, 6.1% very harmful).

**Table 1:** Study sample characteristics according to cannabidiol use (*n* = 1969)

Variable (% missing)	All participants (crude values)	CBD use (crude values)			Prevalence^2^
	*N* (%)	No (*N* = 1772)	Yes (*N* = 197)	*P*-value^1^	% [95% CI]
**Age (mean)** in years **(0)**	51.7 (18.5)	52.8 (18.4)	42.2 (16.7)	< 10^–3^	/
**Age** (in years) **(0)**					
18–24	205 (10.4)	170 (9.6)	35 (17.8)	< 10^–3^	17.1 [12.5 – 22.9]^a^
25–34	278 (14.1)	228 (12.9)	50 (25.4)		18.0 [13.9 – 23.0]^a^
35–49	440 (22.4)	384 (21.7)	56 (28.4)		12.7 [9.9 – 16.2]^a^
50–64	503 (25.6)	469 (26.5)	34 (17.3)		6.7 [4.9 – 9.3]^b^
65–74	291 (14.8)	276 (15.6)	15 (7.6)		5.2 [3.1 – 8.4]^bc^
≥ 75	252 (12.8)	245 (13.8)	7 (3.6)		2.8 [1.3 – 5.7]^bc^
**Gender (0)**					
Men	909 (46.2)	803 (45.3)	106 (53.8)	0.023	11.7 [9.7 – 13.9]
Women	1060 (53.8)	969 (54.7)	91 (46.2)		8.6 [7.0 – 10.4]
**Country of birth (0)**					
France	1858 (94.4)	1670 (94.2)	188 (95.4)	0.493	10.1 [8.8 – 11.6]^a^
Outside France	111 (5.6)	102 (5.8)	9 (4.6)		8.1 [4.2 – 15.0]^a^
**Region (0)**					
Alsace-Champagne-Ardenne-Lorraine	174 (8.8)	148 (8.4)	26 (13.2)	0.327	14.9 [10.4 – 21.1]^a^
Aquitaine-Limousin-Poitou–Charentes	197 (10.0)	180 (10.2)	17 (8.6)		8.6 [5.4 – 13.5]^a^
Auvergne-Rhône-Alpes	236 (12.0)	206 (11.6)	30 (15.2)		12.7 [9.0 – 17.6]^a^
Burgundy-Franche-Comté	85 (4.3)	75 (4.2)	10 (5.1)		11.8 [6.4 – 20.7]^a^
Brittany	115 (5.8)	103 (5.8)	12 (6.1)		10.4 [6.0 – 17.6]^a^
Centre-Val de Loire	89 (4.5)	83 (4.7)	6 (3.0)		6.7 [3.0 – 14.3]^a^
Île-de-France	333 (16.9)	301 (17.0)	32 (16.2)		9.6 [6.9 – 13.3]^a^
Languedoc-Roussillon-Midi-Pyrénées	183 (9.3)	166 (9.4)	17 (8.6)		9.3 [5.8 – 14.5]^a^
Nord-Pas-de-Calais-Picardy	167 (8.5)	154 (8.7)	13 (6.6)		7.8 [4.6 – 13.0]^a^
Normandy	108 (5.5)	102 (5.8)	6 (3.0)		5.6 [2.5 – 11.9]^a^
Pays de la Loire	121 (6.1)	107 (6)	14 (7.1)		11.6 [6.9 – 18.7]^a^
Provence- Alpes-Côte d'Azur	161 (8.2)	147 (8.3)	14 (7.1)		8.7 [5.2 – 14.2]^a^
**City size (0)**					
< 2 000 inhabitants (rural area)	537 (27.3)	488 (27.5)	49 (24.9)	0.839	9.1 [7.0 – 11.9]^a^
2 000—20 000 inhabitants	738 (37.5)	663 (37.4)	75 (38.1)		10.2 [8.2 – 12.6]^a^
20 000—100 000 inhabitants	414 (21.0)	372 (21.0)	42 (21.3)		10.1 [7.6 – 13.5]^a^
> 100 000 inhabitants	280 (14.2)	249 (14.1)	31 (15.7)		11.1 [7.9 – 15.3]^a^
**Socio-professional status (0)**					
Farmer/ craftsperson, trader or business manager/ skilled or unskilled worker	315 (16.0)	270 (15.2)	45 (22.8)	< 10^–3^	14.3 [10.8 – 18.6]^a^
Executive or higher intellectual profession/ Intermediate profession	503 (25.5)	438 (24.7)	65 (33.0)		12.9 [10.3 – 16.2]^a^
Employee	324 (16.5)	285 (16.1)	39 (19.8)		12.0 [8.9 – 16.1]^a^
Pensioner	666 (33.8)	636 (35.9)	30 (15.2)		4.5 [3.2 – 6.4]
Other, no professional activity	161 (8.2)	143 (8.1)	18 (9.1)		11.2 [7.13 – 17.1]^a^
**Educational level (0)**					
No upper secondary school certificate	668 (33.9)	608 (34.3)	60 (30.5)	0.278	9.0 [7.0 – 11.4]^a^
At least upper secondary school certificate	1301 (66.1)	1164 (65.7)	137 (69.5)		10.5 [9.0 – 12.3]^a^
**Dependent children (0)**					
No	1361 (69.1)	1240 (70.0)	121 (61.4)	0.014	8.9 [7.5 – 10.5]
Yes	608 (30.9)	532 (30.0)	76 (38.6)		12.5 [10.1 – 15.4]
**Difficulty paying bills (0)**^**3**^					
Easy	1238 (62.9)	1115 (62.9)	123 (62.4)	0.893	9.9 [8.4 – 11.7]^a^
Difficult	731 (37.1)	657 (37.1)	74 (37.6)		10.1 [8.1 – 12.5]^a^
**Tobacco use (0.4)**					
No	1493 (76.1)	1410 (79.8)	83 (42.6)	< 10^–3^	5.6 [4.5 – 6.8]
Yes	468 (23.9)	356 (20.2)	112 (57.4)		23.9 [20.3 – 28.0]
**Alcohol use (0.3)**^**4**^					
Never	516 (26.3)	471 (26.7)	45 (23.0)	0.074	8.7 [6.6 – 11.5]^a^
Occasional	965 (49.2)	875 (49.5)	90 (45.9)		9.3 [7.6 – 11.3]^a^
Regular	482 (24.6)	421 (23.8)	61 (31.1)		12.7 [10.0 – 15.9]^a^
**Cannabis use (0.5)**					
No	1840 (93.9)	1715 (96.9)	125 (65.8)	< 10^–3^	6.8 [5.7 – 8.0]
Yes	119 (6.1)	54 (3.1)	65 (34.2)		54.6 [45.5 – 63.4]
**Self-reported general health status (0)**^**5**^					
Good	1232 (62.6)	1112 (62.8)	120 (60.9)	0.023	9.7 [8.2 – 11.5]^a^
Quite good	559 (28.4)	510 (28.8)	49 (24.9)		8.8 [6.7 – 11.4]^a^
Poor	178 (9.0)	150 (8.5)	28 (14.2)		15.7 [11.1 – 21.9]
**Chronic disease or health problem (0)**					
No	995 (50.5)	912 (51.5)	83 (42.1)	0.020	8.3 [6.8 – 10.2]^a^
One	712 (36.2)	634 (35.8)	78 (39.6)		11.0 [8.9 – 13.5]^ab^
More than one	262 (13.3)	226 (12.8)	36 (18.3)		13.7 [10.1 – 18.5]^b^
**‘Alternative medicines provide better solutions to health problems than conventional medicines’ (0.3)**					
Disagree	497 (25.3)	456 (25.8)	41 (20.9)	< 10^–3^	8.3 [6.1 – 11.0]^a^
Agree	597 (30.4)	500 (28.3)	97 (49.5)		16.3 [13.5 – 19.4]
No opinion	869 (44.3)	811 (45.9)	58 (29.6)		6.7 [5.2 – 8.5]^a^
**Preferred means to obtain information (0)**					
Television	621 (31.5)	556 (31.4)	65 (33)	0.864	10.5 [8.3 – 13.1]^a^
Radio	211 (10.7)	188 (10.6)	23 (11.7)		10.9 [7.3 – 15.9]^a^
Print media	190 (9.7)	173 (9.8)	17 (8.6)		9.0 [5.6 – 14.0]^a^
Online media	205 (10.4)	185 (10.4)	20 (10.2)		9.8 [6.4 – 14.7]^a^
Other internet ^6^	345 (17.5)	307 (17.3)	38 (19.3)		11.0 [8.1 – 14.8]^a^
Close family members and friends	397 (20.2)	363 (20.5)	34 (17.3)		8.6 [6.2 – 11.8]^a^
**Had heard of CBD (0)**^**7**^					
Had never heard of CBD	605 (30.7)	605 (34.1)	0 (0)	< 10^–3^	/
Only heard of the term ‘CBD’	477 (24.2)	434 (24.5)	43 (21.8)		9.0 [6.8 – 11.9]^a^
Knew a little about CBD	591 (30.0)	539 (30.4)	52 (26.4)		8.8 [6.8 – 11.4]^a^
Had good knowledge of CBD	194 (9.9)	139 (7.8)	55 (27.9)		28.4 [22.4 – 35.1]
Had very good knowledge of CBD	102 (5.2)	55 (3.1)	47 (23.9)		46.1 [36.6 – 55.9]
**‘Do you think that CBD is harmful for health?’ (30.7)**^**7**^					
Did not want to answer	6 (0.4)	5 (0.4)	1 (0.5)	< 10^–3^	16.7 [1.2 – 77.0]^ab^
No opinion	325 (23.8)	308 (26.4)	17 (8.6)		5.2 [3.3 – 8.3]^a^
Not at all	397 (29.1)	286 (24.5)	111 (56.3)		28.0 [23.8 – 32.6]^b^
Slightly harmful	382 (28.0)	342 (29.3)	40 (20.3)		10.5 [7.8 – 13.9]^a^
Quite harmful	171 (12.5)	155 (13.3)	16 (8.1)		9.4 [5.8 – 14.8]^a^
Very harmful	83 (6.1)	71 (6.1)	12 (6.1)		14.5 [8.3 – 23.9]^ab^

*CBD* cannabidiol, *CI* confidence interval

^1^ Chi-squared tests and Student’s t-tests were used in for categorical and continuous variables, respectively

^2^ Common superscript letters denote prevalences not statistically different between modalities. Bonferroni corrections for multiple comparisons were applied

^3^ Very easy or easy vs. difficult or very difficult

^4^ Never vs. occasional (less than once a week or around once a week) vs. regular (several times a week or every day or almost every day)

^5^ Very good or good vs. quite good vs. poor or very poor

^6^ Non-media websites and social networks

^7^ The term ‘CBD’ was used in these questions

### Factors associated with having heard of CBD

After multiple adjustment, having heard of CBD was associated with younger age, being born in France (vs. elsewhere), tobacco use, and cannabis use (Table 2). Conversely, being a farmer/craftsperson/skilled or unskilled labourer, and not having any professional activity were inversely associated with having heard of CBD (vs. people with higher socio-professional occupations).

**Table 2:** Factors associated with having heard of cannabidiol and factors associated with its use (binary logistic regression, multivariable analysis)

Variable	Having heard of cannabidiol (*N* = 1953)		Cannabidiol use (*N* = 1948)	
	aOR [95% CI]	*P*-value	aOR [95% CI]	*P*-value
**Age** (in years)	0.97 [0.96—0.98]	< 10^–3^	0.98 [0.97—0.99]	< 10^–3^
**Country of birth**				
France	1			
Elsewhere	0.55 [0.35 – 0.87]	0.010		
**Socio-professional status**				
Farmer/ craftsperson, trader or business manager/ skilled or unskilled worker	1	**0.022**		
Executive or higher intellectual profession/ Intermediate profession	0.60 [0.43—0.85]	0.004		
Employee	0.81 [0.57—1.16]	0.258		
Pensioner	0.72 [0.49—1.07]	0.108		
Other, no professional activity	0.45 [0.28—0.70]	0.001		
**Tobacco use**				
No	1		1	
Yes	1.38 [1.05—1.82]	0.021	2.82 [1.93—4.11]	< 10^–3^
**Cannabis use**				
No	1		1	
Yes	2.18 [1.14—4.17]	0.019	7.53 [4.66—12.16]	< 10^–3^
**Self-reported general health status **^**1**^				
Good			1	**0.001**
Quite good			1.43 [0.95—2.15]	0.088
Poor			2.68 [1.60—4.49]	< 10^–3^
**‘Alternative medicines provide better solutions to health problems than conventional medicines’**				
Disagree			1	**0.001**
Agree			1.64 [1.03—2.58]	0.035
No opinion			0.81 [0.50—1.32]	0.400

*aOR* adjusted odds ratio, *CI* confidence interval

^1^Very good or good vs. quite good vs. poor and very poor

### Factors associated with CBD use

After multiple adjustment, CBD use was associated with younger age, tobacco use, cannabis use, a poor self-reported general health status (vs. good) and agreement with the statement that alternative medicines provide better solutions to health problems (vs. disagreement) (Table 2).

In post-hoc analyses, running the model without including the alternative medicine statement as an explanatory variable led to similar results. CBD use was associated with age, tobacco and cannabis use and having one or more chronic health problems (data not shown).

### CBD user profiles

After clustering, we retained four clusters, according to the Duda-Hart rule based on a Je(2)/Je (1) ratio of 0.64 and a pseudo T-squared value of 38.8 (Supplementary Fig. 1). Cluster characteristics are given in Table 3.

**Table 3:** Cannabidiol users’ characteristics according to their respective cluster (*n* = 197)

Variable	All participants	Cluster 1^1^ (*n* = 33)	Cluster 2 (*n* = 25)	Cluster 3 (*n* = 71)	Cluster 4 (*n* = 68)
	*N* (%)	*N* (%)	*N* (%)	*N* (%)	*N* (%)
**Age (mean)** (in years)	42.2 (16.7)	59.6 (12.6)	45.8 (14.5)^a^	41.9 (17.7)^a^	32.8 (9.9)
**Gender**					
Men	106 (53.8)	13 (39.4)^a^	22 (88.0)^b^	21 (29.6)^a^	50 (73.5)^b^
Women	91 (46.2)	20 (60.6)	3 (12.0)	50 (70.4)	18 (26.5)
**Country of birth**					
France	188 (95.4)	31 (93.9)^a^	25 (100)^a^	69 (97.2)^a^	63 (92.6)^a^
Elsewhere	9 (4.6)	2 (6.1)	0 (0)	2 (2.8)	5 (7.4)
**Region**					
Alsace-Champagne-Ardenne-Lorraine	26 (13.2)	8 (24.2)^a^	1 (4.0)^a^	10 (14.1)^a^	7 (10.3)^a^
Aquitaine-Limousin-Poitou–Charentes	17 (8.6)	3 (9.1)	3 (12.0)	7 (9.9)	4 (5.9)
Auvergne-Rhône-Alpes	30 (15.2)	7 (21.2)	5 (20.0)	10 (14.1)	8 (11.8)
Burgundy-Franche-Comté	10 (5.1)	0 (0)	3 (12.0)	3 (4.2)	4 (5.9)
Brittany	12 (6.1)	3 (9.1)	2 (8.0)	3 (4.2)	4 (5.9)
Centre-Val de Loire	6 (3.0)	1 (3.0)	1 (4.0)	3 (4.2)	1 (1.5)
Île-de-France	32 (16.2)	1 (3.0)	4 (16.0)	9 (12.7)	18 (26.5)
Languedoc-Roussillon-Midi-Pyrénées	17 (8.6)	2 (6.1)	1 (4.0)	9 (12.7)	5 (7.4)
Nord-Pas-de-Calais-Picardy	13 (6.6)	2 (6.1)	0 (0)	4 (5.6)	7 (10.3)
Normandy	6 (3.0)	1 (3.0)	1 (4.0)	1 (1.4)	3 (4.4)
Pays de la Loire	14 (7.1)	1 (3.0)	3 (12.0)	6 (8.5)	4 (5.9)
Provence-Alpes-Côte d'Azur	14 (7.1)	4 (12.1)	1 (4.0)	6 (8.5)	3 (4.4)
**City size**					
< 2 000 inhabitants (rural area)	49 (24.9)	14 (42.4)^a^	7 (28.0)^ab^	18 (25.4)^ab^	10 (14.7)^b^
2 000—20 000 inhabitants	75 (38.1)	13 (39.4)	10 (40.0)	26 (36.6)	26 (38.2)
20 000—100 000 inhabitants	42 (21.3)	2 (6.1)	6 (24.0)	14 (19.7)	20 (29.4)
> 100 000 inhabitants	31 (15.7)	4 (12.1)	2 (8.0)	13 (18.3)	12 (17.6)
**Type of professional occupation**					
Farmer/ craftsperson, trader or business manager/ skilled or unskilled worker	45 (22.8)	5 (15.2)^a^	8 (32.0)^a^	14 (19.7)^a^	18 (26.5)^a^
Executive or higher intellectual profession/ Intermediate profession	65 (33)	8 (24.2)	10 (40.0)	23 (32.4)	24 (35.3)
Employee	39 (19.8)	6 (18.2)	3 (12.0)	17 (23.9)	13 (19.1)
Pensioner	30 (15.2)	14 (42.4)	1 (4.0)	13 (18.3)	2 (2.9)
Other, no professional activity	18 (9.1)	0 (0)	3 (12.0)	4 (5.6)	11 (16.2)
**Educational level**					
No upper secondary school certificate	60 (30.5)	14 (42.4) ^a^	10 (40.0) ^a^	17 (23.9) ^a^	19 (27.9) ^a^
Upper secondary school certificate	137 (69.5)	19 (57.6)	15 (60.0)	54 (76.1)	49 (72.1)
**Dependent children**					
No	121 (61.4)	23 (69.7) ^a^	20 (80.0) ^a^	39 (54.9) ^a^	39 (57.4) ^a^
Yes	76 (38.6)	10 (30.3)	5 (20.0)	32 (45.1)	29 (42.6)
**Difficulty paying bills**^**2**^					
Easy	123 (62.4)	17 (51.5) ^a^	12 (48.0) ^a^	50 (70.4) ^a^	44 (64.7) ^a^
Difficult	74 (37.6)	16 (48.5)	13 (52.0)	21 (29.6)	24 (35.3)
**Tobacco use**					
No	83 (42.6)	26 (78.8) ^a^	3 (12.0) ^b^	48 (68.6) ^a^	6 (9.0) ^b^
Yes	112 (57.4)	7 (21.2)	22 (88.0)	22 (31.4)	61 (91.0)
**Alcohol use**^**3**^					
Never	45 (23.0)	7 (21.2) ^a^	5 (20.0) ^a^	19 (27.1) ^a^	14 (20.6) ^a^
Occasional	90 (45.9)	16 (48.5)	10 (40.0)	33 (47.1)	31 (45.6)
Regular	61 (31.1)	10 (30.3)	10 (40.0)	18 (25.7)	23 (33.8)
**Cannabis use**					
No	125 (65.8)	33 (100) ^a^	15 (65.2)	69 (100) ^a^	8 (12.3)
Yes	65 (34.2)	0 (0)	8 (34.8)	0 (0)	57 (87.7)
**Self-reported general health status**^**4**^					
Good	120 (60.9)	5 (15.2) ^a^	1 (4.0) ^a^	60 (84.5) ^b^	54 (79.4) ^b^
Quite good	49 (24.9)	18 (54.5)	16 (64.0)	6 (8.5)	9 (13.2)
Poor	28 (14.2)	10 (30.3)	8 (32.0)	5 (7.0)	5 (7.4)
**Chronic disease or health problem**					
No	83 (42.1)	4 (12.1) ^a^	2 (8.0) ^a^	43 (60.6) ^b^	34 (50.0) ^b^
One	78 (39.6)	24 (72.7)	16 (64.0)	19 (26.8)	19 (27.9)
More than one	36 (18.3)	5 (15.2)	7 (28.0)	9 (12.7)	15 (22.1)
**‘Alternative medicines provide better solutions to health problems than conventional medicine’**					
Disagree	41 (20.9)	8 (24.2) ^a^	15 (60.0)	9 (12.7) ^a^	9 (13.4) ^a^
Agree	97 (49.5)	5 (15.2)	2 (8.0)	43 (60.6)	47 (70.1)
No opinion	58 (29.6)	20 (60.6)	8 (32.0)	19 (26.8)	11 (16.4)
**Preferred means to obtain information**					
Television	65 (33.0)	17 (51.5) ^a^	11 (44.0) ^a^	18 (25.4) ^a^	19 (27.9) ^a^
Radio	23 (11.7)	4 (12.1)	3 (12.0)	10 (14.1)	6 (8.8)
Print media	17 (8.6)	2 (6.1)	1 (4.0)	3 (4.2)	11 (16.2)
Online media	20 (10.2)	2 (6.1)	3 (12.0)	7 (9.9)	8 (11.8)
Other internet^5^	38 (19.3)	4 (12.1)	3 (12.0)	19 (26.8)	12 (17.6)
Close family members and friends	34 (17.3)	4 (12.1)	4 (16.0)	14 (19.7)	12 (17.6)
**Cannabidiol use**					
Less than once a week	100 (50.8)	15 (45.5) ^a^	15 (60.0) ^a^	37 (52.1) ^a^	33 (48.5) ^a^
Around once a week	32 (16.2)	5 (15.2)	1 (4.0)	8 (11.3)	18 (26.5)
Several times a week	34 (17.3)	7 (21.2)	2 (8.0)	15 (21.1)	10 (14.7)
Every day or almost every day	31 (15.7)	6 (18.2)	7 (28.0)	11 (15.5)	7 (10.3)
**‘Do you think that CBD is harmful for health?’**					
Did not want to answer	1 (0.5)	0 ^a^	0 ^a^	1 (1.4) ^a^	0 ^a^
No opinion	17 (8.6)	7 (21.2)	5 (19.2)	3 (4.2)	2 (2.9)
Not at all harmful	111 (56.3)	18 (54.5)	16 (61.5)	50 (70.4)	27 (39.1)
Slightly harmful	40 (20.3)	6 (18.2)	4 (15.4)	8 (11.3)	22 (31.9)
Quite harmful	16 (8.1)	2 (6.1)	0	4 (5.6)	10 (14.5)
Very harmful	12 (6.1)	0	0	5 (7.0)	7 (10.1)

^1^ Common superscript letters denote prevalences not statistically different between clusters. Bonferroni corrections for multiple comparisons were applied

^2^ Very easy or easy vs. difficult or very difficult

^3^ Never vs. occasional (less than once a week or around once a week) vs. regular (several times a week or every day or almost every day)

^4^ Very good or good vs. quite good vs. poor or very poor

^5^ Non-media websites and social networks

The typical individual from Cluster 1 (*n* = 33) was older, lived in a rural setting, did not smoke either tobacco or cannabis, had one chronic health condition, and had no opinion about alternative medicines. The typical individual from Cluster 2 (*n* = 25) was male, had difficulties paying bills, consumed alcohol regularly, had one chronic health problem, and disagreed with the alternative medicines statement. The typical individual from Cluster 3 (*n* = 71) was an educated mother who had no difficulty paying bills, had a good self-reported health status, and agreed with the alternative medicines statement. The typical individual from Cluster 4 (*n* = 68) was a young man who smoked both tobacco and cannabis, and who agreed with the alternative medicines statement. CBD use frequency poorly discriminated between clusters (Table 3).

In the sub-sample of CBD users (*n* = 197) and in the set of variables used for the AHC, cannabis use was correlated with gender, age and tobacco use (*p* < 0.05). Age was correlated with tobacco use and self-reported health status (*p* < 0.05).

### Factors associated with CBD perceived harmfulness

After multiple adjustment, younger age, being born in France (vs. elsewhere), and cannabis use were all associated with both perceiving CBD as harmful and harmless (vs. no opinion on CBD harmfulness), while being a woman and regular alcohol use were associated with perceiving CBD as harmless (vs. no opinion). Having no opinion about the alternative medicines statement (vs. disagreeing with it) was associated with having no opinion on CBD harmfulness. Finally, print media and online news sites (vs. television) as the preferred media to obtain information were, respectively, negatively and positively associated with perceiving CBD as harmless, while other internet websites (including social networks) were associated with perceiving CBD as harmful (Table 4).

**Table 4:** Factors associated with perceiving cannabidiol as harmful or not (multinomial logistic regression, with ‘no opinion’ as reference)

Variables	Cannabidiol is not harmful^1^ (*n* = 397)		Cannabidiol is harmful^1^ (*n* = 636)	
	aRRR [95% CI]	*P*-value	aRRR [95% CI]	*P*-value
**Age** (in years)	0.98 [0.97—0.99]	< 10^–3^	0.99 [0.98 – 1.00]	0.007
**Gender**				
Men	1		1	
Women	1.40 [1.01—1.94]	0.044	0.90 [0.67—1.19]	0.453
**Country of birth**				
France	1		1	
Elsewhere	0.43 [0.22—0.87]	0.018	0.53 [0.29—0.95]	0.032
**Alcohol use**^**2**^				
Never	1	**0.002**	1	**0.151**
Occasional	1.19 [0.82—1.72]	0.368	1.13 [0.81—1.57]	0.479
Regular	2.25 [1.41—3.57]	0.001	1.51 [0.99—2.30]	0.057
**Cannabis use**				
No	1		1	
Yes	8.21 [2.80—24.06]	< 10^–3^	5.52 [1.95—15.65]	0.001
**‘Alternative medicines provide better solutions to health problems than conventional medicines’**				
Disagree	1	** < 10**^**–3**^	1	**0.001**
Agree	1.26 [0.81—1.95]	0.301	1.21 [0.81—1.81]	0.351
No opinion	0.57 [0.38—0.84]	0.005	0.64 [0.45—0.92]	0.015
**Preferred means to obtain information**				
Television	1	**0.005**	1	**0.017**
Radio	1.33 [0.79—2.24]	0.284	1.21 [0.74—1.97]	0.443
Print media	0.47 [0.26—0.86]	0.014	0.65 [0.40—1.07]	0.088
Online media	1.80 [1.04—3.10]	0.035	1.58 [0.95—2.62]	0.079
Other internet^3^	1.47 [0.93—2.33]	0.098	1.53 [1.00—2.33]	0.049
Close family members and friends	1.10 [0.69—1.73]	0.691	1.42 [0.95—2.12]	0.089

*aRRR* adjusted relative risk ratio, *CI* confidence interval

^1^ Not at all harmful vs. a little harmful, quite harmful, and very harmful

^2^ Never vs. occasional (less than once a week or around once a week) vs. regular (several times a week or every day or almost every day)

^3^ Non-media websites and social networks

Reclassifying ‘slightly harmful’ as ‘harmless’ (sensitivity analysis) only marginally altered the significance of a few response modalities (Supplementary Table 2).

Post-hoc analysis revealed that in the sub-sample of people who had heard of CBD, those who used it were more likely than non-users to perceive CBD as harmless (56.6% vs. 24.6%, respectively; *p* < 10^–3^).

## Discussion

In a national sample of adults in France, the prevalence of CBD use was 10%, while 30% had never heard of it. CBD use was associated with younger age, tobacco use, cannabis use, poor self-reported general health status, and a perception that alternative medicines provide better solutions to health problems than conventional medicines. Cluster analysis revealed four different user profiles based on socio-demographics and behavioural characteristics. Those who perceived CBD as harmless were more likely to use it.

To our knowledge, the present study is the first to assess the national prevalence of CBD use in France. The prevalence of CBD use in France is higher than in Germany (lifetime use of non-prescribed CBD was estimated at 4.3%, and current use at 1.1% [24]) but lower than in the US and Canada ( 26.1 and 16.2%, respectively [23]). These higher prevalences are partly explained by the fact that cannabis is more accessible in the US and Canada than in France. The survey in the US and Canada also found a positive association between cannabis use and CBD use. Similarly to other studies, our results confirm that a substantial proportion of those aware of CBD-based products did not have an opinion with regards to their harmfulness, and few perceived them as harmful [6, 23].

We did not find any association between CBD use and gender, educational level, and the difficulty to pay bills. However, we found an association with socio-professional category.

The CBD market is relatively new. Thirty percent of our adult population in France had never heard of CBD. In contrast, half the population was aware of CBD products in Germany [24]. As the recent increased visibility and popularity of CBD is likely to continue in adults—and especially in persons with health conditions—we can expect that consumption prevalence will also increase in the coming years.

To our knowledge, our study is the first to describe the profile of CBD users in the French general population. Clusters differed in terms of age, gender, city size, tobacco and cannabis use, health characteristics and their view on the alternative medicines statement (Table 3). Our cluster analysis confirmed the relationship between cannabis and CBD use, suggesting that cannabis co-users constitute a distinct category of CBD users [26, 30, 31]. Given results from human studies and observational data, we can hypothesise that some people use CBD in an attempt to reduce their cannabis consumption [31, 32]. We also found that poor self-reported general health status was associated with a higher likelihood of using CBD, and that health characteristics differ between clusters, suggesting CBD is used for therapeutic reasons [17]. Therapeutic use was frequently reported in a self-selected convenience sample of CBD users mainly from the US [16], and in a Swiss study [33]. Nevertheless, we cannot draw definitive conclusions about CBD’s impact on various health conditions—including anxiety, sleeping disorders and chronic pain [8]—which are often cited as reasons for its use [6, 16, 17, 18, 34], and which are often described as beneficial on internet sites, often for marketing purposes [35, 36]. The frequency of CBD use did not differ between clusters (daily use between 10 and 28%). However, since we did not collect information on CBD routes of administration and dosages, we are unable to determine whether or not there is a difference in CBD intake between clusters. Further studies are needed to explore such differences between users, as well as possible related stigma (e.g. using CBD-rich cannabis to smoke vs. sublingual drops).

Given the association we found between CBD use and agreeing with the alternative medicines statement, it would seem that CBD was perceived as a safe alternative to conventional medicine to maintain health and prevent disease [6], a perception probably driven by its plant-based origin. However, answers from Clusters 1 and 2 highlighted that not all CBD users considered alternatives medicines superior.

The sizeable proportion (30.8%) of our study sample who had not heard of CBD highlights that a substantial part of the French population is still unaware of it, and its potential properties. In respondents who had heard of it, 57.4% perceived it to be not at all or slightly harmful. This reflects a generally positive attitude toward CBD and its safety profile, and is consistent with previous findings [6, 37] and scientific data [3, 4]. In Goodman et al.’s study, approximately 30% of responders perceived CBD as ‘good’ or ‘very good’ for health, and 25% as ‘neither good nor bad’. Furthermore, in that study, 30% replied ‘do not know’ to the question on CBD harmfulness, which is very close to the 29.3% in our study who had no opinion.

As expected, we found a negative correlation between perceived harmfulness of CBD and CBD use [23]. This relationship has already been highlighted for cannabis [20, 38] and for psychoactive substances more generally [39]. Importantly, we found that both cannabis and regular alcohol use were associated with positive and negative perceptions of CBD safety. It would therefore seem that compared to non-substance users, substance users more often had enough information and/or felt concerned enough about CBD to be able to form their own opinion about its safety.

Our results from this first assessment of CBD use prevalence in France have several implications. First, the fact that 10% of the study population declared using CBD makes the regulation of CBD-based products in France—and EU in general—an urgent issue, especially in light of reports of adulteration and mislabelling [12, 14, 40], as well as misinformation [1, 36] and drug-testing issues [41]. Second, we identified different profiles of users so creating segmented markets may help consumers find the most suitable CBD-based products for their specific needs. Third, the relatively high prevalence of CBD use in our study highlights the need for reliable information on CBD’s properties, and the importance of raising people’s and physicians’ awareness of possible adverse events (e.g., CBD hepatotoxicity at very high doses [15]) and drug–drug interactions with CBD [42]. Finally, as CBD is already used by French cannabis users, and given that it may lower harms related to illegal cannabis use (i.e., THC-rich cannabis from the black market) [31, 43], the incorporation of CBD-based products into harm reduction approaches for cannabis users could be considered.

The main strengths of the present study are the sampling methodology used based on quotas, the fact that older adults were included unlike in previous CBD use studies, and that willingness to participate was not related to CBD use (as it was not presented as a primary objective). Another strength is that we simultaneously assessed awareness, use, and perception of CBD, and highlighted the relationships between them. Finally, profiling CBD users shed more light on specific sub-groups of users, and emphasized that factors associated with CBD use are differentially distributed across sub-groups.

The study also has limitations. While online surveys may reduce desirability bias (as compared to face-to-face interview), there may have been sampling bias (excluding people without internet or using it marginally). Moreover, since our study was cross-sectional, we were not able to explore the relationship between perceived harmfulness and CBD use. Further studies are needed to test whether perceived absence of harmfulness is prior or subsequent to CBD use. As the survey did not primarily focus on CBD use, only a few questions were asked about it. Therefore, data regarding the patterns of use (doses, route of administration, duration of use) and reasons for using CBD were not collected. These would have been valuable to better cluster users. Finally, as the sample size was based on the quota method, the number of CBD users was small, which limited the power of our analyses.

To conclude, 10% of the adult population in our French sample reported using CBD, and several user profiles emerged. Further research in the general population on the knowledge, the perceived effectiveness and the motivation for CBD consumption, as well as on its real-life toxicity are required. Our results indirectly advocate clearer European regulations on CBD to ensure that people have access to safe and high-quality products. Moreover, more accurate and accessible information about CBD’s effects and possible interactions with other drugs is needed. Finally, repeated surveys on CBD use in the general population are needed to monitor its use and clarify the reasons for using it.

## Supplementary Information

**Additional file 1: Supplementary Table 1. **Characteristics of included vs. excluded participants.

**Additional file 2: Supplementary Table 2. **Factors associated with perceiving cannabidiol as harmful or not, sensitivityanalysis (multinomial logistic regression, with ‘no opinion’ as reference).

**Additional file 3: Supplementary Figure 1.** Dendrogram of the cluster analysis of users.

## Data Availability

The datasets generated and/or analyzed during the current study are not publicly available due to ongoing data treatment but are available from the corresponding author on reasonable request.

## Abbreviations

CBD: Cannabidiol
THC: Δ9-Tetrahydrocannabinol
CI: Confidence interval
IQR: Interquartile range
