# Supplementary material for: Cannabidiol use and perceptions in France: a national survey
Source: BMC Public Health. 2022 Aug 29;22:1628. doi: 10.1186/s12889-022-14057-0 (PMC9421113; doi:10.1186/s12889-022-14057-0)
Supplement: Supplementary file 1 — Additional file 1: Supplementary Table 1. Characteristics of included vs. excluded participants. [file 12889_2022_14057_MOESM1_ESM.docx]

**Supplementary Table 1: Characteristics of included vs. excluded participants**

| **Variable (% missing)** | **All participants**  **N = 2022** | **Excluded participants (N = 53)** | **Included participants (N =1969)** |  | **Included participants**  **(N =1969), weighted values^2^** |
| --- | --- | --- | --- | --- | --- |
|  | N (%) | N (%) | N (%) | P-value^1^ | % [95% CI] |
| **Age (mean)** in years **(0)** | 51.5 (18.6) | 43.9 (19.1) | 51.7 (18.5) | 0.003 | - |
| **Gender  (0)** |  |  |  |  |  |
| Men | 932 (46.1) | 23 (43.4) | 909 (46.2) | 0.028 | 47.6 [45.4 – 49.9] |
| Women | 1089 (53.9) | 29 (54.7) | 1060 (53.8) |  | 52.4 [50.1 – 54.7] |
| Other | 1 (0) | 1 (1.9) | 0 (0) |  |  |
| **Country of birth (0)** |  |  |  |  |  |
| France | 1908 (94.4) | 50 (94.3) | 1858 (94.4) | 1.000 | 94.0 [92.8 – 95.0] |
| Elsewhere | 114 (5.6) | 3 (5.7) | 111 (5.6) |  | 6.0 [5.0 – 7.2] |
| **Region (0)** |  |  |  |  |  |
| Alsace-Champagne-Ardenne-Lorraine | 181 (9.0) | 7 (13.2) | 174 (8.8) | 0.357 | 8.6 [7.4 – 9.9] |
| Aquitaine-Limousin-Poitou-Charentes | 202 (10.0) | 5 (9.4) | 197 (10.0) |  | 9.5 [8.3 – 10.9] |
| Auvergne-Rhône-Alpes | 242 (12.0) | 6 (11.3) | 236 (12.0) |  | 12.4 [11.0 – 14.0] |
| Burgundy-Franche-Comté | 91 (4.5) | 6 (11.3) | 85 (4.3) |  | 4.4 [3.6 – 5.4] |
| Brittany | 118 (5.8) | 3 (5.7) | 115 (5.8) |  | 5.2 [4.3 – 6.2] |
| Centre-Val de Loire | 89 (4.4) | 0 (0) | 89 (4.5) |  | 3.9 [3.2 – 4.8] |
| Île-de-France | 341 (16.9) | 8 (15.1) | 333 (16.9) |  | 18.6 [16.9 – 20.5] |
| Languedoc-Roussillon-Midi-Pyrénées | 189 (9.3) | 6 (11.3) | 183 (9.3) |  | 9.4 [8.2 – 10.8] |
| Nord-Pas-de-Calais-Picardy | 173 (8.6) | 6 (11.3) | 167 (8.5) |  | 9.1 [7.8 – 10.5] |
| Normandy | 109 (5.4) | 1 (1.9) | 108 (5.5) |  | 5.1 [4.2 – 6.1] |
| Pays de la Loire | 123 (6.1) | 2 (3.8) | 121 (6.1) |  | 5.9 [4.9 – 7.0] |
| Provence-Alpes-Côte d'Azur | 164 (8.1) | 3 (5.7) | 161 (8.2) |  | 7.9 [6.8 – 9.2] |
| **City size (0)** |  |  |  |  |  |
| < 2 000 inhabitants (rural area) | 557 (27.5) | 20 (37.7) | 537 (27.3) | 0.411 | 26.5 [24.6 – 28.5] |
| 2 000 - 20 000 inhabitants | 755 (37.3) | 17 (32.1) | 738 (37.5) |  | 38.4 [36.2 – 40.6] |
| 20 000 - 100 000 inhabitants | 424 (21.0) | 10 (18.9) | 414 (21.0) |  | 21.5 [19.7 – 23.4] |
| > 100 000 inhabitants | 286 (14.1) | 6 (11.3) | 280 (14.2) |  | 13.6 [12.2 – 15.2] |
| **Socio-professional status** **(0)** |  |  |  |  |  |
| Farmer/ craftsperson, trader or business manager/ skilled or unskilled worker | 323 (16.0) | 8 (15.1) | 315 (16.0) | 0.021 | 15.3 [13.8 – 17.0] |
| Executive or higher intellectual profession/ Intermediate profession | 516 (25.5) | 13 (24.5) | 503 (25.5) |  | 24.5 [22.7 – 26.5] |
| Employee | 333 (16.5) | 9 (17.0) | 324 (16.5) |  | 14.3 [12.9 – 15.8] |
| Pensioner | 678 (33.5) | 12 (22.6) | 666 (33.8) |  | 33.8 [31.7 – 36.0] |
| Other, no professional activity | 172 (8.5) | 11 (20.8) | 161 (8.2) |  | 12.1 [10.5 – 14.0] |
| **Educational level (0)** |  |  |  |  |  |
| No upper secondary school certificate | 694 (34.3) | 26 (49.1) | 668 (33.9) | 0.022 | 34.2 [32.0 – 36.3] |
| Upper secondary school certificate | 1328 (65.7) | 27 (50.9) | 1301 (66.1) |  | 65.8 [63.7 – 68.0] |
| **Dependent children (0)** |  |  |  |  |  |
| No | 1399 (69.2) | 38 (71.7) | 1361 (69.1) | 0.688 | 68.5 [66.3 – 70.5] |
| Yes | 623 (30.8) | 15 (28.3) | 608 (30.9) |  | 31.5 [29.5 – 33.7] |
| **Difficulty paying bills (0)^3^** |  |  |  |  |  |
| Easy | 1258 (62.2) | 20 (37.7) | 1238 (62.9) | < 10^-3^ | 62.8 [60.5 – 64.9] |
| Difficult | 764 (37.8) | 33 (62.3) | 731 (37.1) |  | 37.2 [35.1 – 39.5] |
| **Tobacco use (0.4)** |  |  |  |  |  |
| No | 1529 (76.1) | 36 (75.0) | 1493 (76.1) | 0.855 | 76.1 [74.1 – 78.0] |
| Yes | 480 (23.9) | 12 (25.0) | 468 (23.9) |  | 23.9 [22.0 – 25.9] |
| **Alcohol use (0.3)^4^** |  |  |  |  |  |
| Never | 533 (26.5) | 17 (37.8) | 516 (26.3) | 0.033 | 26.7 [24.7 – 28.7] |
| Occasional | 989 (49.3) | 24 (53.3) | 965 (49.2) |  | 48.6 [46.3 – 50.9] |
| Regular | 486 (24.2) | 4 (8.9) | 482 (24.6) |  | 24.7 [22.9 – 26.7] |
| **Cannabis use (0.5)** |  |  |  |  |  |
| No | 1883 (93.9) | 43 (91.5) | 1840 (93.9) | 0.491 | 93.6 [92.3 – 94.6] |
| Yes | 123 (6.1) | 4 (8.5) | 119 (6.1) |  | 6.4 [5.4 – 7.7] |
| **Self-reported general health status (0)^5^** |  |  |  |  |  |
| Good | 1263 (62.5) | 31 (58.5) | 1232 (62.6) | 0.661 | 62.7 [60.5 – 64.9] |
| Quite good | 577 (28.5) | 18 (34.0) | 559 (28.4) |  | 28.1 [26.1 – 30.1] |
| Poor | 182 (9.0) | 4 (7.5) | 178 (9.0) |  | 9.2 [8.0 – 10.7] |
| **Chronic disease or health problem (0)** |  |  |  |  |  |
| No | 1027 (50.8) | 32 (60.4) | 995 (50.5) | 0.217 | 50.9 [48.6 – 53.1] |
| One | 725 (35.9) | 13 (24.5) | 712 (36.2) |  | 35.9 [33.8 – 38.1] |
| More than one | 270 (13.4) | 8 (15.1) | 262 (13.3) |  | 13.3 [11.8 – 14.9] |
| **‘Alternative medicines provide better solutions to health problems than conventional medicine’** **(0.3)** |  |  |  |  |  |
| Disagree | 503 (25.0) | 6 (12.2) | 497 (25.3) | 0.038 | 25.6 [23.7 – 27.6] |
| Agree | 619 (30.8) | 22 (44.9) | 597 (30.4) |  | 30.5 [28.4 – 32.6] |
| No opinion | 890 (44.2) | 21 (42.9) | 869 (44.3) |  | 43.9 [41.7 – 46.2] |
| **Preferred means to obtain information (0)** |  |  |  |  |  |
| Television | 638 (31.6) | 17 (32.1) | 621 (31.5) | 0.099 | 31.7 [29.6 – 33.8] |
| Radio | 213 (10.5) | 2 (3.8) | 211 (10.7) |  | 10.6 [9.3 – 12.0] |
| Print media | 193 (9.5) | 3 (5.7) | 190 (9.6) |  | 9.6 [8.4 – 11.0] |
| Online media | 207 (10.2) | 2 (3.8) | 205 (10.4) |  | 10.7 [9.4 – 12.2] |
| Other internet^6^ | 359 (17.8) | 14 (26.4) | 345 (17.5) |  | 17.4 [15.8 – 19.2] |
| Close family members and friends | 412 (20.4) | 15 (28.3) | 397 (20.2) |  | 20.0 [18.3 – 21.9] |

^1^ Chi-squared tests and Student t-tests were used for categorical and continuous variables, respectively.

^2^ To counterbalance the over- or under-representation of specific population categories with respect to gender, age, socio-professional status, and population density in the region of residence, weighting factors derived from the National Institute of Statistics and Economic Studies data were used.

^3^ Very easy or easy vs. difficult or very difficult

^4^ Never vs. occasional (less than once a week or around once a week) vs. regular (several times a week or every day or almost every day)

^5^ Very good or good vs. quite good vs. poor or very poor

^6^ Non-media websites and social networks

CI, confidence interval
