# Supplementary material for: Cannabidiol use and perceptions in France: a national survey
Source: BMC Public Health. 2022 Aug 29;22:1628. doi: 10.1186/s12889-022-14057-0 (PMC9421113; doi:10.1186/s12889-022-14057-0)
Supplement: Supplementary file 2 — Additional file 2: Supplementary Table 2. Factors associated with perceiving cannabidiol as harmful or not, sensitivityanalysis (multinomial logistic regression, with ‘no opinion’ as reference). [file 12889_2022_14057_MOESM2_ESM.docx]

**Supplementary Table 2: Factors associated with perceiving cannabidiol as harmful or not, sensitivity analysis (multinomial logistic regression, with ‘no opinion’ as reference)**

| **Variable** | **Cannabidiol is harmless^1^**  **(n = 779)** | | **Cannabidiol is harmful^1^**  **(n = 254)** | |
| --- | --- | --- | --- | --- |
|  | aRRR [95% CI] | P-value | aRRR [95% CI] | P-value |
| **Age** (in years) | 0.99 [0.98 - 0.99] | < 10^-3^ | 0.99 [0.98 – 1.00] | 0.007 |
| **Gender** |  |  |  |  |
| Men |  |  |  |  |
| Women |  |  |  |  |
| **Country of birth** |  |  |  |  |
| France | 1 |  | 1 |  |
| Elsewhere | 0.46 [0.26 - 0.81] | 0.007 | 0.58 [0.27 - 1.21] | 0.148 |
| **Tobacco use** |  |  |  |  |
| No | 1 |  | 1 |  |
| Yes | 1.13 [0.81 - 1.59] | 0.462 | 0.61 [0.39 - 0.95] | 0.028 |
| **Alcohol use^2^** |  |  |  |  |
| Never | 1 | **0.011** | 1 | **0.366** |
| Occasional | 1.20 [0.86 - 1.66] | 0.280 | 1.07 [0.71 - 1.59] | 0.753 |
| Regular | 1.84 [1.23 - 2.76] | 0.003 | 1.41 [0.85 - 2.32] | 0.180 |
| **Cannabis use** |  |  |  |  |
| No | 1 |  | 1 |  |
| Yes | 6.38 [2.22 - 18.32] | 0.001 | 6.38 [2.04 - 19.93] | 0.001 |
| **‘Alternative medicines provide better solutions to health problems than conventional medicine’** |  |  |  |  |
| Disagree | 1 | **< 10^-3^** | 1 | **0.004** |
| Agree | 1.29 [0.87 - 1.92] | 0.205 | 1.03 [0.64 - 1.67] | 0.891 |
| No opinion | 0.63 [0.44 - 0.89] | 0.009 | 0.57 [0.37 - 0.88] | 0.011 |
| **Preferred means to obtain information** |  |  |  |  |
| Television | 1 | **0.001** | 1 | **0.270** |
| Radio | 1.32 [0.83 - 2.12] | 0.245 | 1.04 [0.58 - 1.88] | 0.885 |
| Print media | 0.60 [0.37 - 0.97] | 0.039 | 0.53 [0.28 - 1.01] | 0.053 |
| Online media | 1.75 [1.07 - 2.87] | 0.026 | 1.33 [0.73 - 2.43] | 0.353 |
| Other internet^3^ | 1.72 [1.14 - 2.61] | 0.010 | 1.08 [0.65 - 1.82] | 0.761 |
| Close family members and friends | 1.34 [0.90 – 2.00] | 0.145 | 1.16 [0.71 - 1.89] | 0.547 |

^1^ Not at all or slightly harmful vs. quite or very harmful

^2^ Never vs. occasional (less than once a week or around once a week) vs. regular (several times a week or every day or almost every day)

^3^ Non-media websites and social networks

aRRR, adjusted relative risk ratio
