# Supplementary figures and images for: Cannabidiol use and perceptions in France: a national survey
Source: BMC Public Health. 2022 Aug 29;22:1628. doi: 10.1186/s12889-022-14057-0 (PMC9421113; doi:10.1186/s12889-022-14057-0)

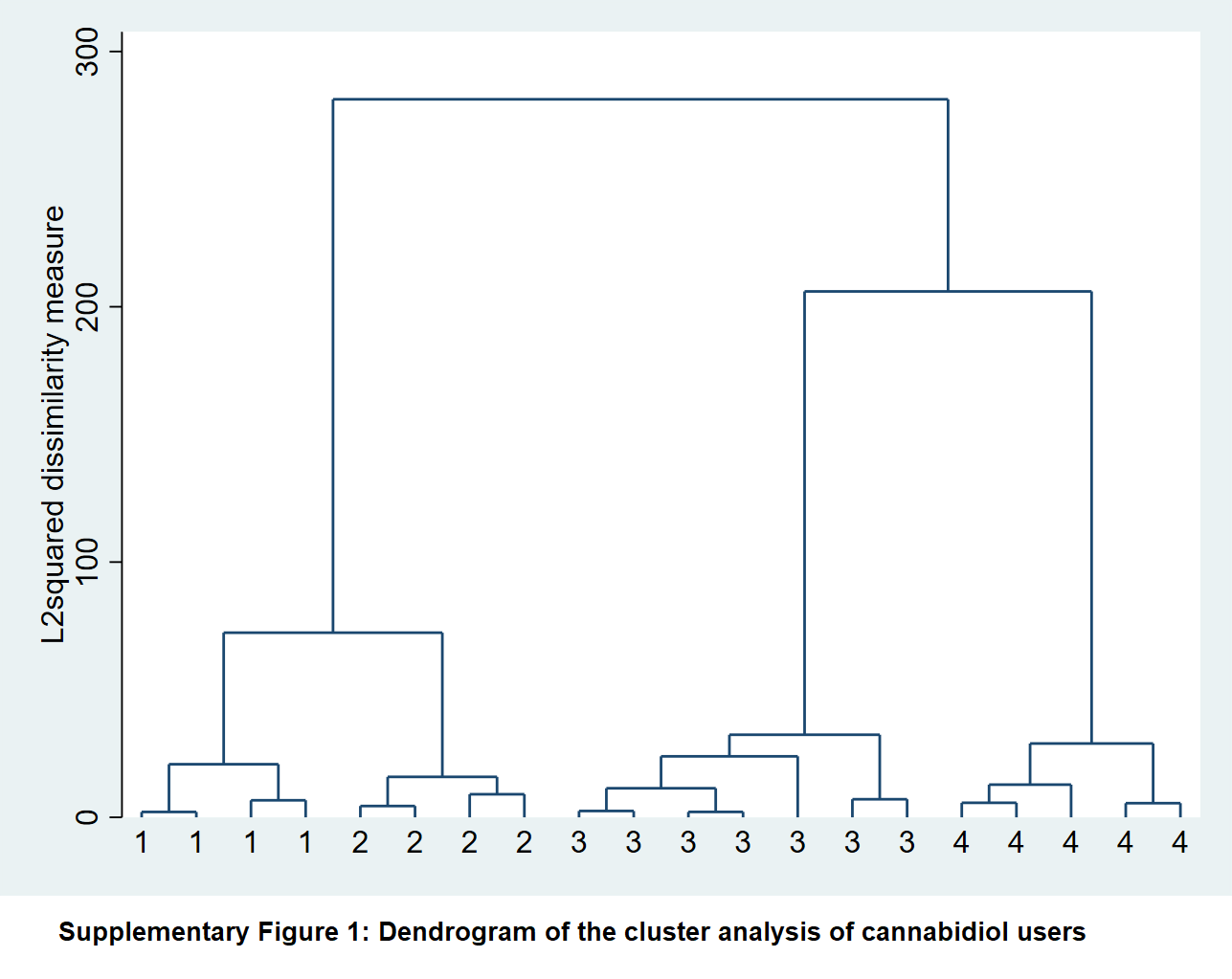

Supplement: Supplementary file 3 — Additional file 3: Supplementary Figure 1. Dendrogram of the cluster analysis of users. [file 12889_2022_14057_MOESM3_ESM.png]
